# Mechanochemically Engineered CaO–CeO_2_ Dual-Function Catalysts for Sustainable Glycerol Carbonate Production without Solvents

**DOI:** 10.1021/acs.energyfuels.5c01580

**Published:** 2025-06-19

**Authors:** Patcharaporn Inrirai, Runzhe Yu, Daniel Goma Jiménez, Nancy Artioli, Haresh Manyar

**Affiliations:** † School of Chemistry and Chemical Engineering, 1596 Queen’s University Belfast, David-Keir Building, Belfast BT9 5AG, U.K.; ‡ Department of Civil, Environmental, Architectural Engineering and Mathematics, University of Brescia, Via Branze, 43, Brescia 25123, Italy

## Abstract

Upgrading biorefinery-derived waste such as glycerol to fuel-additives and high-value products is essential to further enhance the productivity, profitability, and circularity of the biorefinery concept to achieve a green and sustainable net-zero world. This study explores the catalytic conversion of glycerol into glycerol carbonate using calcium oxide–cerium oxide (CaO–CeO_2_) dual-function catalytic materials. Herein, a clean and efficient approach was developed to synthesize CaO–CeO_2_ materials using a green mechanochemical method and then utilize these as catalyst in sustainable and solvent-free synthesis of glycerol carbonate to enhance the circular economy of biorefineries while reducing their carbon footprint. The catalysts were comprehensively characterized using XRD, FTIR, ICP, N_2_ sorption, CO_2_-TPD, and SEM/EDS analyses and evaluated for their catalytic activity. Among the catalysts studied, 40 wt % CaO–CeO_2_ exhibited the highest catalytic activity, achieving 95% glycerol conversion and 99% selectivity to glycerol carbonate under optimized conditions (10 wt % catalyst loading relative to glycerol, 90 °C, 60 min, and a glycerol/ DMC molar ratio of 1:3). This catalyst showed excellent reusability, maintaining high conversion over four cycles. The transesterification reaction followed irreversible second-order reaction kinetics with an activation energy of 46.9 kJ mol^–1^. The synergistic interplay between the basic sites of the Ca^2+^–O^2–^ pair and the oxygen vacancies in the CeO_2_ matrix at the CaO–CeO_2_ interface work in tandem to enhance the catalytic activity for glycerol carbonate production. We have developed a highly efficient, cost-effective, and environment-friendly approach for the sustainable production of glycerol carbonate from glycerol.

## Graphical abstract

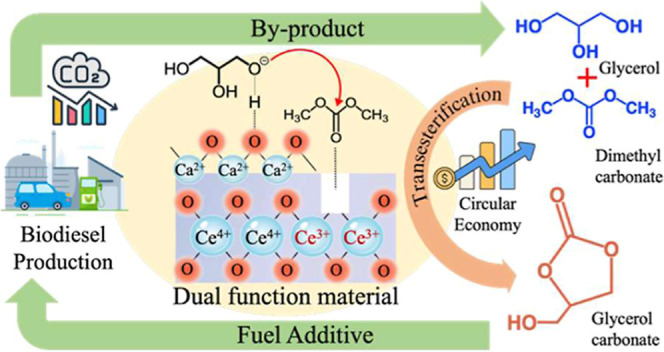

## 1 Introduction

The increasing global demand for energy has intensified environmental challenges, particularly due to greenhouse gas emissions, with carbon dioxide (CO_2_) being a major contributor. Biodiesel is widely recognized as a sustainable alternative to fossil fuels, offering significant reductions in CO_2_, carbon monoxide (CO), sulfur oxides (SO_*x*_), particulate matter (PM), and volatile organic compounds (VOCs) emissions.^1^

Global biodiesel production has doubled from 20 million tons in 2010 to 40 million tons in 2022, with projections indicating stable production levels in the near future.^2,3^ However, the biodiesel industry generates glycerol as a major byproduct, accounting for approximately 10% of the total production by weight. Although often considered waste, glycerol has substantial potential for value-added applications across various industries, including fuel additives, polymer synthesis, hydrogen production, fuel cells, and pharmaceuticals. Our research group has keen interest in upgrading glycerol into high-value products for advancing circular economy.^4–8^ One of the promising strategies for glycerol valorization is its conversion to glycerol carbonate via transesterification. Glycerol carbonate is regarded as an excellent fuel additive due to the capability to increase the fuel octane number and oxidation stability and diminish particle emissions. By upgrading glycerol to glycerol carbonate, we can not only minimize the carbon footprint of biorefineries but also improve the economic profitability as the selling price of crude glycerol is US$210 to $390/t, while that of glycerol carbonate is around US$3000/ton. Glycerol carbonate exhibits excellent physicochemical properties, such as a low freezing point (−69 °C), high boiling point (137–140 °C), high density (1.4 g mL^–1^ at 25 °C), and a high dielectric constant (115).^9^ Additionally, it is nontoxic, highly soluble in water, and biocompatible. Owing to its carbonyl and hydroxyl functional groups, glycerol carbonate has diverse applications, particularly as a high-value chemical intermediate for polymer production, as well as in solvents, surfactants, lubricants, cosmetics, and fuel additives.^7^

Existing research on glycerol transesterification has predominantly focused on homogeneous catalytic systems. However, homogeneous catalysts present challenges related to catalyst cost, postreaction separation, and complex product purification. To overcome these issues, heterogeneous catalysts have been explored as viable alternatives. Calcium oxide (CaO) was first introduced as a catalyst for glycerol transesterification with dimethyl carbonate by Ochoa-Gómez et al.,^10^ who compared its catalytic performance with other basic materials such as magnesium oxide (MgO) and calcium carbonate (CaCO_3_). The catalytic efficiency of CaO was significantly influenced by calcination temperature, achieving a 91% glycerol carbonate yield under optimized conditions. Similarly, Simanjuntak et al.^11^ reported a CaO-catalyzed process with a 94% conversion and high selectivity to glycerol carbonate. However, catalyst deactivation occurred due to the formation of calcium glycerate complexes. To enhance the stability and reusability of CaO, researchers have explored its incorporation into supports, such as activated alumina. Lu et al.^12^ demonstrated that CaO supported on activated alumina extrudates, synthesized using polyacrylamide as a pore-forming agent, exhibited superior stability compared to unsupported CaO, with the yield decreasing from 91% to 63% after six reaction cycles. Modifying CaO with basic dopants has been shown to improve both its activity and stability. For instance, doping CaO with potassium nitrate has been shown to enhance catalytic performance compared to pure CaO.^13^ As a result, the development of efficient and stable catalysts remains a key research focus for the transesterification of glycerol to glycerol carbonate.

While cerium oxide (CeO_2_) is generally not considered highly active in transesterification reactions, it has been identified as a beneficial component for enhancing the basicity of solid base catalysts.^14^ Parameswaram et al.^15^ reported that CeO_2_ exhibits remarkable catalytic performance when modified with other metal oxides, leading to improved stability. Similarly, Niu et al.^16^ found that introducing cerium into CaO/MgO catalysts enhances their stability by preventing the leaching of active CaO species into the reaction medium, thereby improving catalyst reusability.^17^ CeO_2_, a fluorite-type material, possesses oxygen vacancy defects and an inherent oxygen storage capacity (OSC),^18^ both of which contribute to its catalytic efficiency. The ability of Ce cations to transition between Ce^3+^ and Ce^4+^ states facilitates electronic conductivity and lattice expansion. These unique properties make CeO_2_ an effective promoter and support material in various catalytic applications.^19^

Beyond catalytic performance, factors such as separation efficiency, environmental impact, and cost-effectiveness play significant roles in catalyst development. A key advantage of CaO is its derivation from abundant and renewable natural sources, often from waste materials, aligning with the principles of a circular economy.^20,21^ In this study, CaO–CeO_2_ composites synthesized through greener mechanochemical methods have gained considerable attention. By optimizing the morphology and electronic properties of CaO–CeO_2_, the interaction between these two components can be fine-tuned to enhance oxygen vacancy formation, thereby promoting the catalytic reaction via sorption-enhanced catalysis. Moreover, CaO–CeO_2_ materials provide a cost-effective and scalable solution, making them highly attractive for industrial applications.

## 2 Experimental Section

### 2.1 Materials

All chemicals were acquired from reputable manufacturers and used without further purification. Cerium­(IV) oxide (CeO_2_, 99.95%) and ethanol (C_2_H_5_OH, >99.8%) were purchased from Sigma-Aldrich. Glycerol (HOCH_2_CH­(OH)­CH_2_OH, >99%), dimethyl carbonate (C_3_H_6_O_3_, 99%), *p*-xylene (C_8_H_10_, 99%), and calcium oxide (CaO, 99.9%) were obtained from Alfa Aesar.

### 2.2 Catalyst Preparation

Supported calcium oxide (CaO) catalysts were prepared using a green mechanochemical method using cogrinding.^4,22^ The required amount of CaO was mixed with CeO_2_ and ground for 15 min. The resulting catalyst was then sieved using a 125 μm mesh and dried at 80 °C overnight.

### 2.3 Catalyst Characterization

The prepared catalysts were characterized using N_2_ physisorption, elemental analysis, X-ray diffraction (XRD), Fourier-transform infrared spectroscopy (FTIR), thermogravimetric analysis (TGA), CO_2_-temperature-programmed desorption (CO_2_-TPD), scanning electron microscopy (SEM), and energy-dispersive X-ray spectroscopy (EDX). Surface area and porosity were analyzed using N_2_ adsorption/desorption, with the surface area calculated by the Brunauer–Emmett–Teller (BET) method, while the pore volume and pore diameter were determined using the Barrett–Joyner–Halenda (BJH) method. Metal loadings of the catalysts were measured by inductively coupled plasma optical emission spectrometry (ICP-OES) by using a PerkinElmer Optima 4300 instrument. XRD patterns were recorded by using Cu Kα radiation (λ = 0.15418 nm) on a Panalytical instrument equipped with reflection geometry. The scattered intensities were collected over a wide-angle range of 5° to 80° (2θ) with a step size of 0.02°. FTIR spectra were obtained using an Agilent Technologies Cary 630 FTIR spectrometer in the 4000–450 cm^–1^ range with an ATR sampling module. TGA profiles were recorded using a Q5000 thermogravimetric analyzer (TA Instruments) to study the decomposition behavior of the catalysts under airflow conditions, with a temperature ramp of 10 °C min^–1^ to 900 °C. The basicity of the prepared catalysts was determined using CO_2_-TPD analysis. The CO_2_-TPD measurements were carried out on a Micromeritics MicroActive AutoChem II 2920 system, using approximately 0.2 g of catalyst powder. The catalyst samples were first heated under a helium flow to 500 °C at a rate of 10 °C min^–1^ for 60 min. After cooling to 50 °C, a 2% CO_2_ in helium mixture was passed over the sample. The sample was then heated from 50 to 950 °C at a rate of 10 °C min^–1^ under helium flow. SEM images were recorded using an FEI Quanta FEG Environmental SEM to examine the catalyst morphology. In addition to imaging, EDX mapping was conducted. The EDX analysis was performed on a relatively even surface of a catalyst particle to determine the average surface density of the corresponding metal atoms.

### 2.4 Reaction Procedure and Analysis Method

Catalytic activity tests were performed in a 100 mL glass reactor equipped with a magnetic stirring bar and a condenser. The reactor was mechanically agitated and maintained in an isothermal oil bath at the desired temperature. In a typical reaction, glycerol (GLY), dimethyl carbonate (DMC), and the catalyst were added to the glass reactor and stirred at 800 rpm for 1 h.^4^ The reaction analysis was carried out using a Clarus 500 PerkinElmer gas chromatograph equipped with a Zebron ZB-Wax column and *p*-xylene as an internal standard. For reusability studies, the catalysts were separated from the reaction mixture by centrifugation, washed with ethanol, dried overnight at 80 °C, and then calcined at 800 °C for 3 h.^20^

Glycerol conversion was calculated according to eq 1, where *N*_A0_ and *N*_At_ represent the initial moles of glycerol and the moles at a given time *t*, respectively.

1 $$Conversion\;\left(\%\right)=\left(\frac{N_{A0}-N_{At}}{N_{A0}}\right)\times 100$$

Furthermore, the selectivity of the desired product was calculated using eq 2, where *A*_d_ represents the area under the curve for the desired product and the sum *A*_i_ considers the total area of all products formed in the reaction.

2 $$Selectivity\;\left(\%\right)=\left(\frac{A_{d}}{A_{i}}\right)\times 100$$

## 3 Results and Discussion

### 3.1 Catalyst Characterization

The XRD patterns of the prepared catalysts are shown in Figure 1a. The diffraction peaks observed at 2θ values of 28.3°, 33.1°, 47.4°, 56.2°, 59.2°, 69.5°, 76.6°, and 79.0° correspond to the (111), (200), (220), (311), and (222) planes of the pure fluorite cubic structure of CeO_2_.^23^ The characteristic diffraction peaks corresponding to CaO are (111), (200), (220), (311), (222), and (400).^23^ The incorporation of CaO into the CeO_2_ matrix resulted in negligible changes to the XRD patterns of CeO_2_. The CaO phase exhibited relatively low-intensity peaks, while strong reflections corresponding to the fluorite-type cubic structure of CeO_2_ were identified in the XRD pattern.^24^ Upon doping 15 wt % CaO onto CeO_2_, additional diffraction peaks of CaO emerged at 2θ values of 23° and 37°, with their intensity increasing as the CaO content in the catalyst increased.

**Figure 1:**
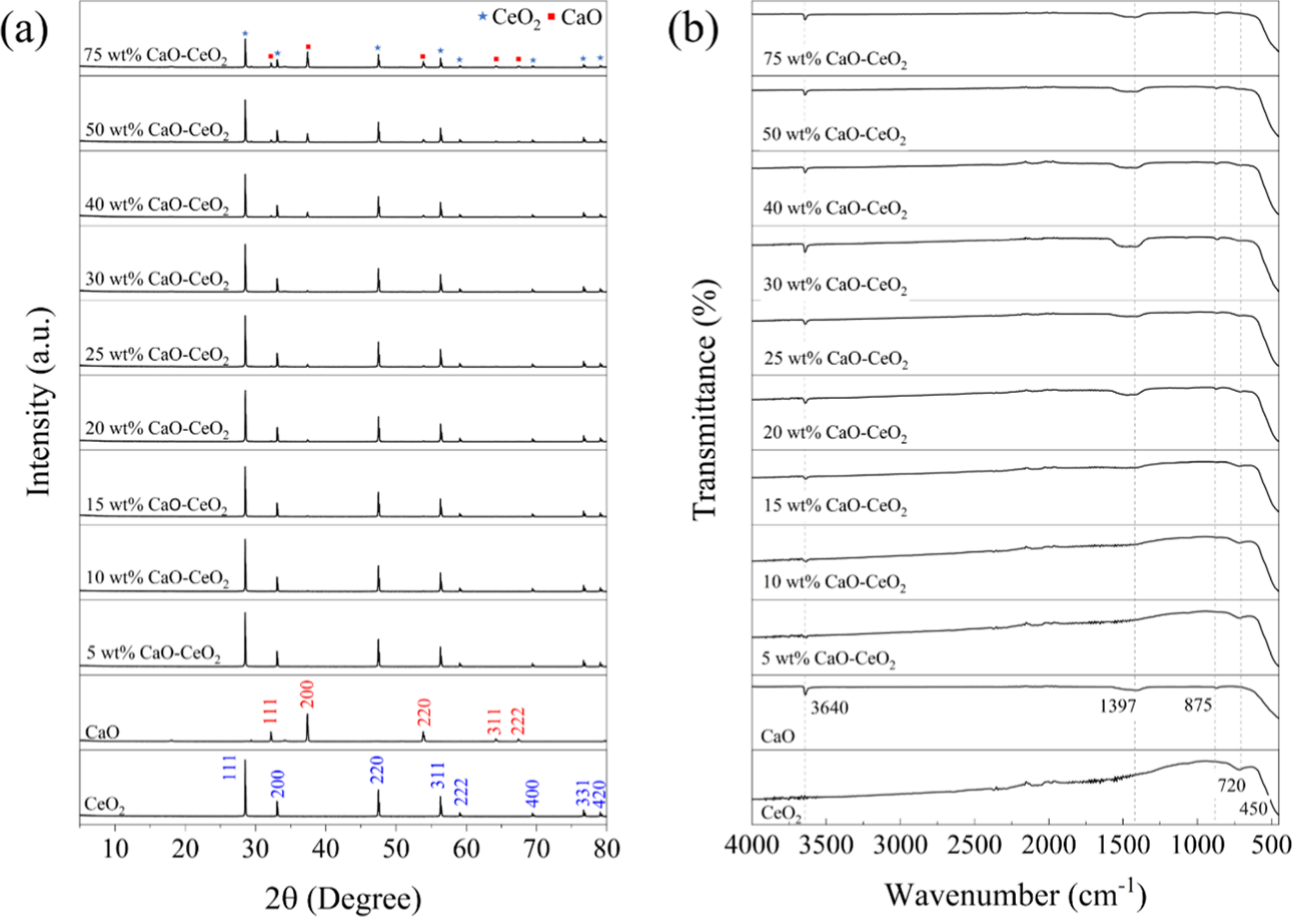
(a) X-ray diffraction patterns of CeO_2_, CaO, and 5–75 wt % of CaO–CeO_2_ catalysts, and (b) FTIR spectra of CeO_2_, CaO, and 5–75 wt % of CaO–CeO_2_ catalysts.

The FTIR spectra of CaO, CeO_2_, and CaO–CeO_2_ catalysts are shown in Figure 1b. All spectra exhibit a broad absorption band around 450 cm^–1^, attributed to Ca–O, Ce–O, and Ca–O–Ce stretching vibrations.^24^ The peak at 875 cm^–1^ corresponds to the out-of-plane stretching vibrations of the carbonate group (CO_3_). The band at 1397 cm^–1^ is characteristic of asymmetric C–O stretching in the carbonate group. The two bands around 1571 cm^–1^ can be assigned to different C–H vibrational modes. The band observed around 3640 cm^–1^ corresponds to the stretching of hydroxyl groups from hydroxide species.^23,25^

The total basicity of the prepared catalysts was evaluated using temperature-programmed desorption of CO_2_, with the quantified basicity values presented in Table 1. Pure CaO exhibited a basicity of 0.577 mmol g^–1^, whereas pure CeO_2_ showed no detectable basic sites on its surface. However, upon doping CeO_2_ with CaO, the basicity of the catalysts increased progressively from 0.131 mmol g^–1^ to 0.540 mmol g^–1^, as the CaO content increased from 5 to 40 wt %. When the CaO content exceeded 50 wt % in the prepared catalysts, the total basicity continued to increase, surpassing that of pure CaO. A basicity of 0.739 mmol g^–1^ was observed for the 75 wt % CaO–CeO_2_ catalyst, which could be attributed to the synergistic interaction between CaO and CeO_2_.^26^ The TPD-CO_2_ profiles of pure CeO_2_, CaO, and CaO-doped CeO_2_ catalysts are presented in Figure 2, showing desorption behavior from 100 to 800 °C. Desorption peaks below 200 °C indicate the presence of weak basic sites, while peaks in the 200–570 °C range are related to moderate basic sites. The peaks at high temperatures correspond to the strong basic sites.^27^ Pure CeO_2_ showed no detectable basic sites on its surface, whereas the CaO catalyst exhibited both moderate and strong basic sites. With 5–10 wt % CaO incorporated into CeO_2_, moderate basic sites were observed. As the CaO content increased, the enhanced basicity of the prepared catalysts led to a shift toward strong basic sites. 50 and 75 wt % CaO–CeO_2_ catalysts exhibited a higher density of strong basic sites compared to pure CaO. The total basicity values of all catalysts, shown in Table 1, align well with the TCD signals.

**Table 1:** BET Surface Area, Pore Volume, Average Pore Diameter, and Total Basicity of the Prepared Catalysts

catalyst	BET surface area (m^2^ g^–1^)	pore volume (cm^3^ g^–1^)	average pore diameter (nm)	total basicity (mmol g^–1^)
CeO_2_	2.46	0.0063	11.78	0.000
5 wt % CaO–CeO_2_	2.71	0.0074	12.53	0.131
10 wt % CaO–CeO_2_	2.28	0.0071	19.41	0.135
15 wt % CaO–CeO_2_	2.35	0.0075	13.52	0.234
20 wt % CaO–CeO_2_	2.00	0.0067	20.34	0.245
25 wt % CaO–CeO_2_	2.18	0.0079	18.46	0.335
30 wt % CaO–CeO_2_	2.80	0.0110	18.30	0.452
40 wt % CaO–CeO_2_	2.50	0.0092	15.23	0.540
50 wt % CaO–CeO_2_	3.25	0.0132	16.72	0.593
75 wt % CaO–CeO_2_	3.15	0.0142	18.56	0.739
CaO	1.80	0.0085	21.80	0.577

**Figure 2:**
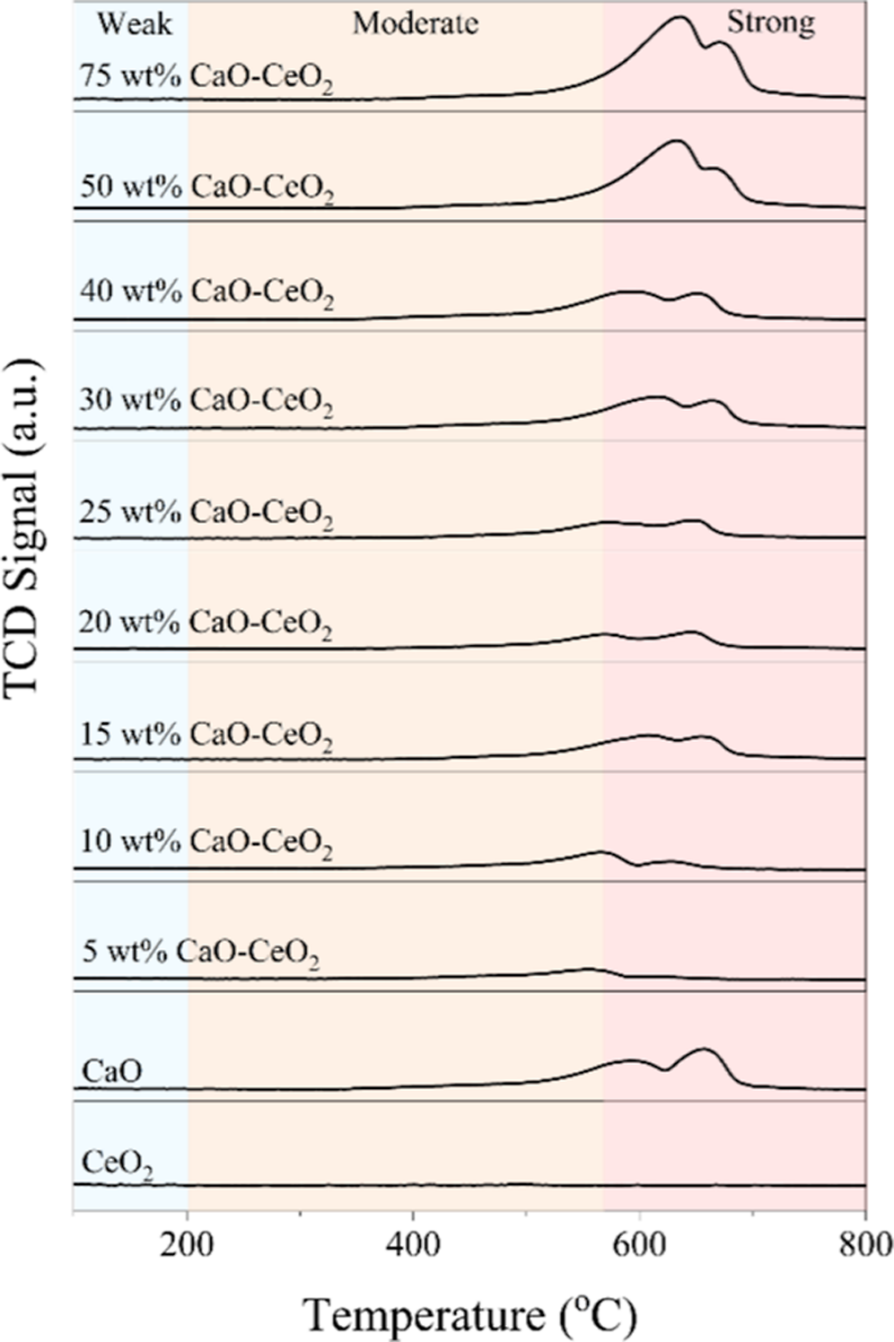
CO_2_-TPD profiles of the prepared catalysts.

Figure 3 presents the adsorption–desorption isotherms of the catalysts. The hysteresis loops observed for CeO_2_, CaO, and 40 wt % CaO–CeO_2_ correspond to type IV hysteresis loops, as classified by the IUPAC, indicating the presence of mesoporous structures.^23^

**Figure 3:**
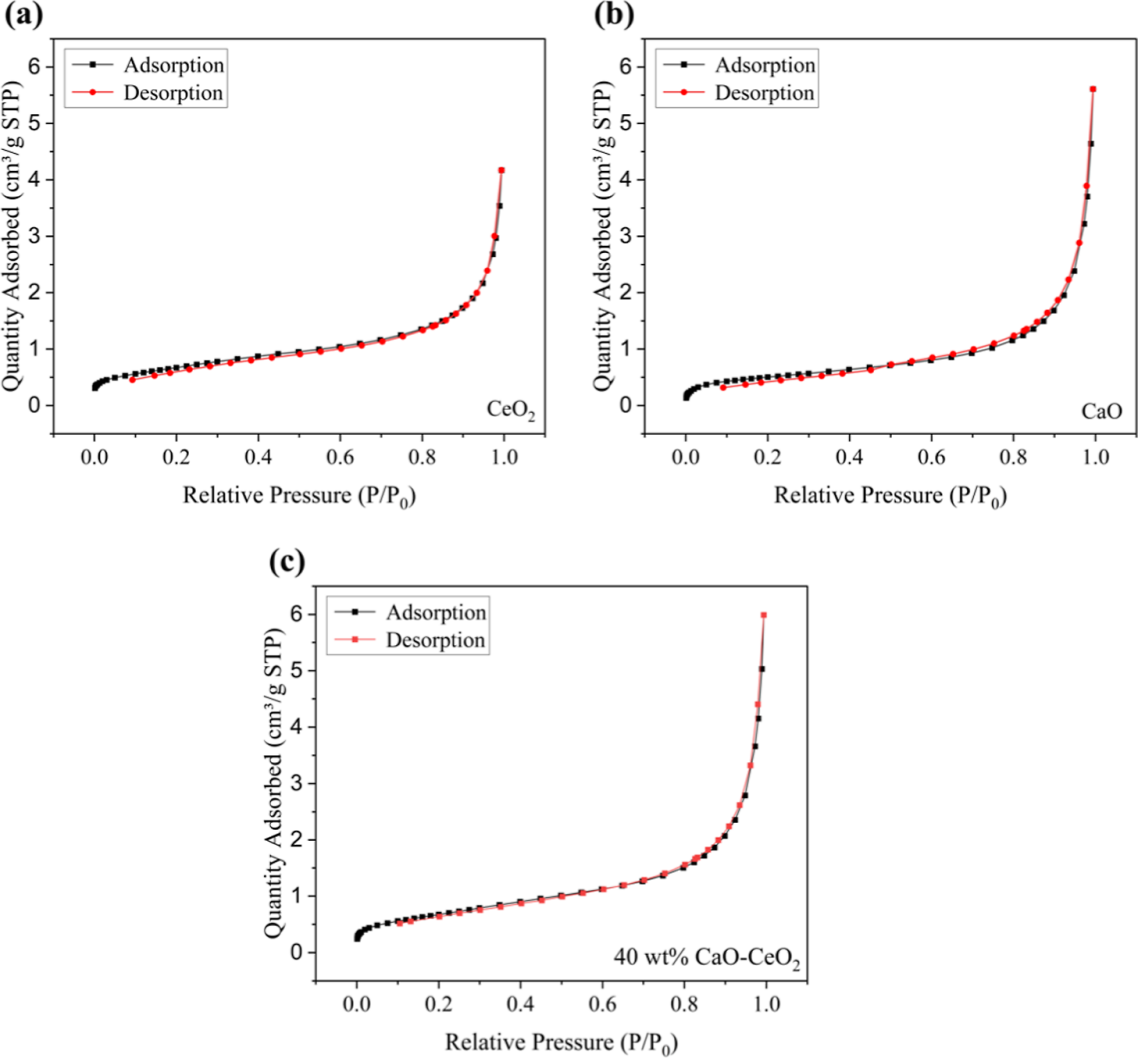
N_2_ adsorption/desorption isotherms of CaO–CeO_2_ mixed oxide materials: ((a) CeO_2_; (b) CaO; and (c) 40 wt % CaO–CeO_2_).

The TGA results, shown in Figure 4, demonstrate that the CeO_2_ catalyst exhibited excellent thermal stability, with no significant weight loss over the evaluated temperature range. In contrast, the CaO catalyst exhibited a notable weight loss of approximately 11% at 400 °C, which was attributed to the decomposition of Ca­(OH)_2_. A second weight loss stage of about 6% was observed between 600 and 700 °C, corresponding to the thermal conversion of CaCO_3_ to CaO. The 40 wt % CaO–CeO_2_ catalyst exhibited a similar two-step weight loss pattern to that of CaO, with a total mass loss of 13%. However, the final residue was higher than that of pure CaO, suggesting that the CeO_2_ support enhances the thermal stability of CaO. The weight loss trends observed in the TGA measurements align well with the decomposition behavior of the Ca–Ce oxide catalysts previously reported by Kingkam et al.^23^

**Figure 4:**
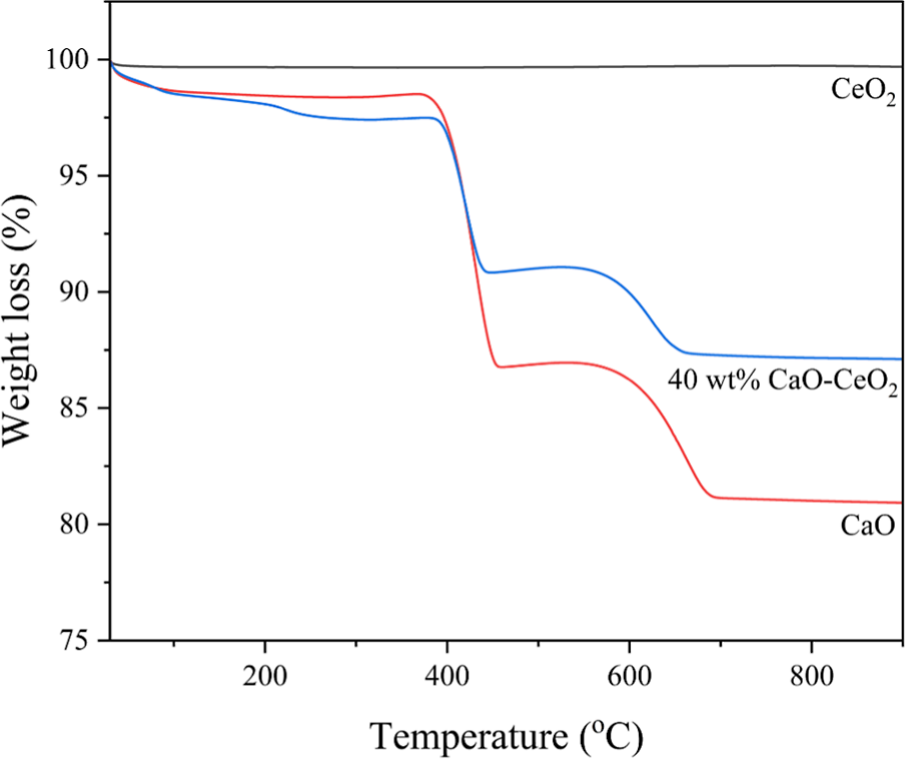
Thermogravimetric analysis (TGA) curves of CeO_2_, CaO, and 40 wt % CaO–CeO_2_.

Table 2 summarizes the mass percentage calculations based on the metal concentrations (mg kg^–1^) obtained from ICP analysis. The measured weight percentages of Ca and Ce in the catalysts closely matched the expected theoretical ratio, indicating the successful incorporation of CaO onto the CeO_2_ matrix.

**Table 2:** ICP Analysis of the Prepared Catalysts

catalyst	target CaO content (wt %)	actual CaO content (wt %)	target CeO_2_ content (wt %)	actual CeO_2_ content (wt %)
5 wt % CaO–CeO_2_	5.00	3.74	95.00	96.26
10 wt % CaO–CeO_2_	10.00	8.47	90.00	91.53
15 wt % CaO–CeO_2_	15.00	12.45	95.00	87.55
20 wt % CaO–CeO_2_	20.00	17.02	80.00	82.98
25 wt % CaO–CeO_2_	25.00	19.94	75.00	80.06
30 wt % CaO–CeO_2_	30.00	27.63	70.00	72.37
40 wt % CaO–CeO_2_	40.00	35.35	60.00	65.65
50 wt % CaO–CeO_2_	50.00	45.06	50.00	54.94
75 wt % CaO–CeO_2_	75.00	71.61	25.00	28.39

The BET surface area, pore volume, and average pore diameter of all catalysts synthesized in this study are summarized in Table 1. The BET surface area is a critical parameter that indicates the available surface area for catalytic activity. CeO_2_ showed a BET surface area of 2.46 m^2^ g^–1^, which was higher than that of CaO (1.8 m^2^ g^–1^). However, CaO exhibited a greater pore volume and average pore diameter than pure CeO_2_. Following the incorporation of CaO on CeO_2_, the BET surface area ranged from 2.00 to 3.25 m^2^ g^–1^, while both the pore volume and average pore diameter increased with CaO doping. The average pore diameter varied between 12.53 and 20.34 nm, which is significantly larger than the molecular diameters of the reactants, glycerol (0.65 nm) and dimethyl carbonate (0.55 nm), while the molecular diameter of the product, glycerol carbonate, is 0.52 nm.^21^ Ideally for optimal catalytic performance, the catalyst should possess a pore diameter considerably larger than these molecular dimensions, typically within the mesoporous range (2–50 nm), to facilitate effective diffusion and minimize steric hindrance. However, excessively large pores may reduce the surface area available for catalytic activity.

The morphologies of the catalysts were analyzed using SEM, as shown in Figure 5. Pure CeO_2_ exhibited a predominantly spherical structure with a uniform distribution and smaller particle size than CaO (Figure 5a). In contrast, pure CaO appeared as agglomerates of irregularly shaped crystalline particles, as shown in Figure 5b. After mechanical grinding, the particle size of CaO was reduced and became more evenly dispersed on the CeO_2_ catalyst. The elemental mapping of the 40 wt % CaO–CeO_2_ catalyst (Figure 5f) showed a homogeneous distribution of Ce and Ca oxides, suggesting a possibility of strong interactions between the two metal oxides. When comparing the distribution of Ca and Ce, the mapping revealed a scattered distribution of each element. In Figure 5f, blue specks represent Ca atoms, while yellow specks indicate Ce atoms, showing that Ce is present in significantly higher amounts than Ca. This observation is further supported by the elemental spectrum, which quantifies the weight percentage of each component. The measured Ca–Ce weight percentage ranged from 23% to 78%, aligning well with the expected composition.

**Figure 5:**
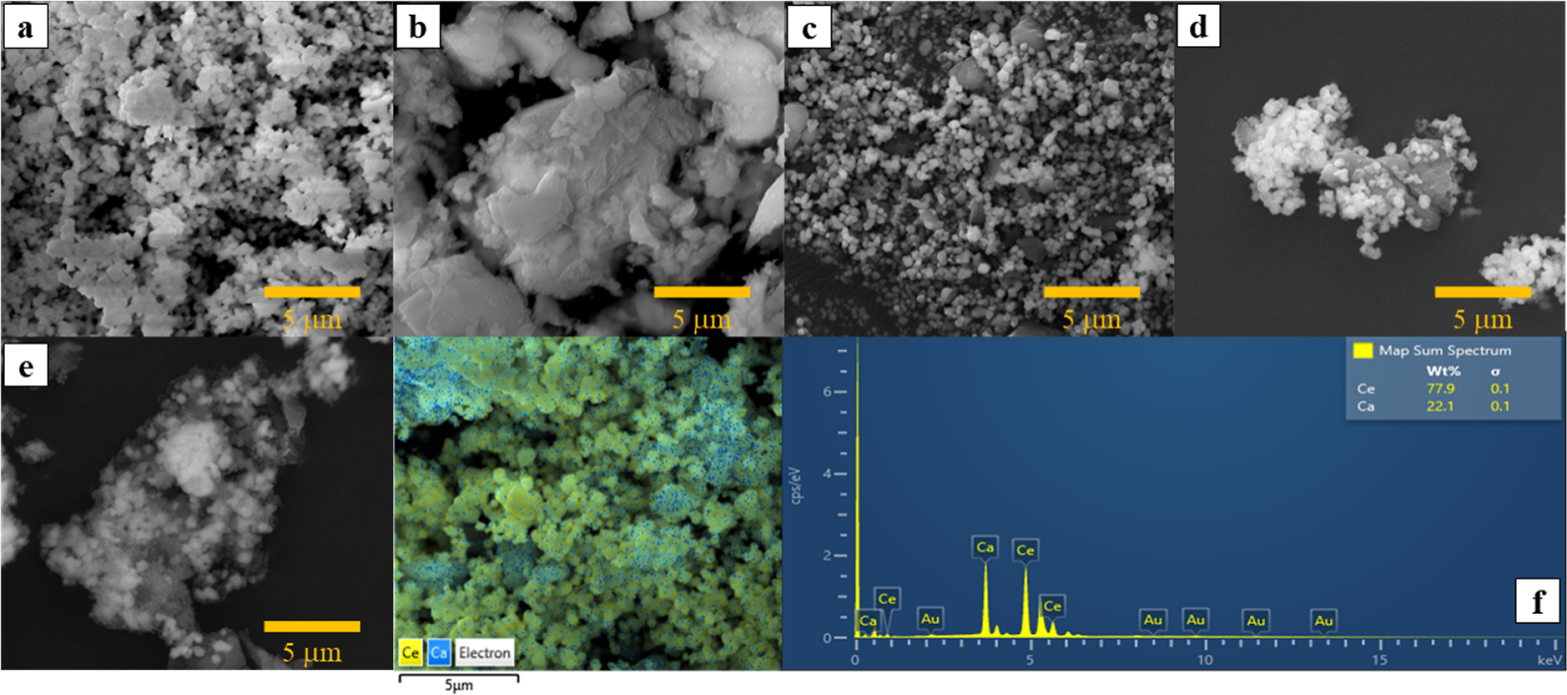
SEM images of (a) CeO_2_, (b) CaO catalyst, (c) 40 wt % CaO–CeO_2_, (d) used 40 wt % CaO–CeO_2_ (calcined at 800 °C), and (e) used 40 wt % CaO–CeO_2_ and (f) EDX mapping of 40 wt % CaO–CeO_2_.

### 3.2 Effect of Different Contents of CaO Doped on CeO_2_

To optimize the yield of glycerol carbonate (GC), CeO_2_ was doped with varying amounts of calcium oxide (CaO) using a mechanical grinding method. The catalytic performance of the CaO-doped CeO_2_ catalysts was evaluated under typical reaction conditions: a catalyst loading of 10 wt %, a reactant molar ratio of 1:3 (GLY: DMC), reaction temperature 90 °C, and reaction time 1 h. As shown in Figure 6, the highest GC yield of 95% was achieved using 40 wt % CaO doped on CeO_2_ as catalyst. The catalytic activity of CeO_2_ increased with increasing CaO content, with the GC yield improving progressively from 5 wt % to 40 wt % CaO loading and then similar GC yields for CeO_2_ catalysts doped with 50 and 75 wt % CaO. Based on these findings, 40 wt % CaO–CeO_2_ was selected for further optimization studies to maximize the GC yield.

**Figure 6:**
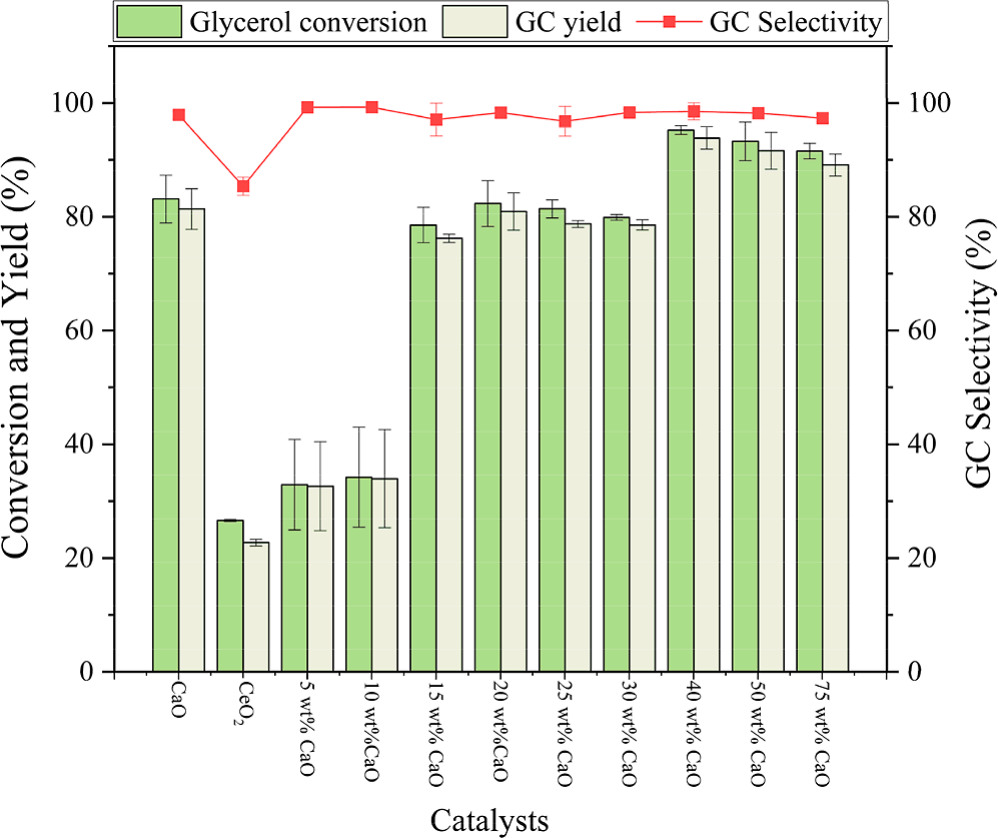
Glycerol conversion, glycerol carbonate selectivity, and yield of prepared CaO, CeO_2_, and 5–75 wt % CaO–CeO_2_ catalysts.

### 3.3 Effect of Catalyst Loading

The transesterification reaction is influenced by the presence of active sites on the basic catalyst, which depends on both the type and amount of metal loading on its surface. A higher number of active sites enhances reactant interactions, thereby improving the conversion efficiency.^28^ To determine the optimal catalyst loading for the maximum GC yield, reactions were performed by varying the catalyst loading from 5 to 20 wt % (relative to glycerol). As shown in Figure 7a, the highest GC yield of 94% was achieved using the catalyst loading of 10 wt %. Increasing the catalyst loading beyond 10 wt % did not result in a significant improvement in yield, with a 20 wt % catalyst loading achieving a GC yield of 93%. Therefore, a catalyst loading of 10 wt % was identified as suitable for further parameter optimization in the transesterification reaction.^20,29^

**Figure 7:**
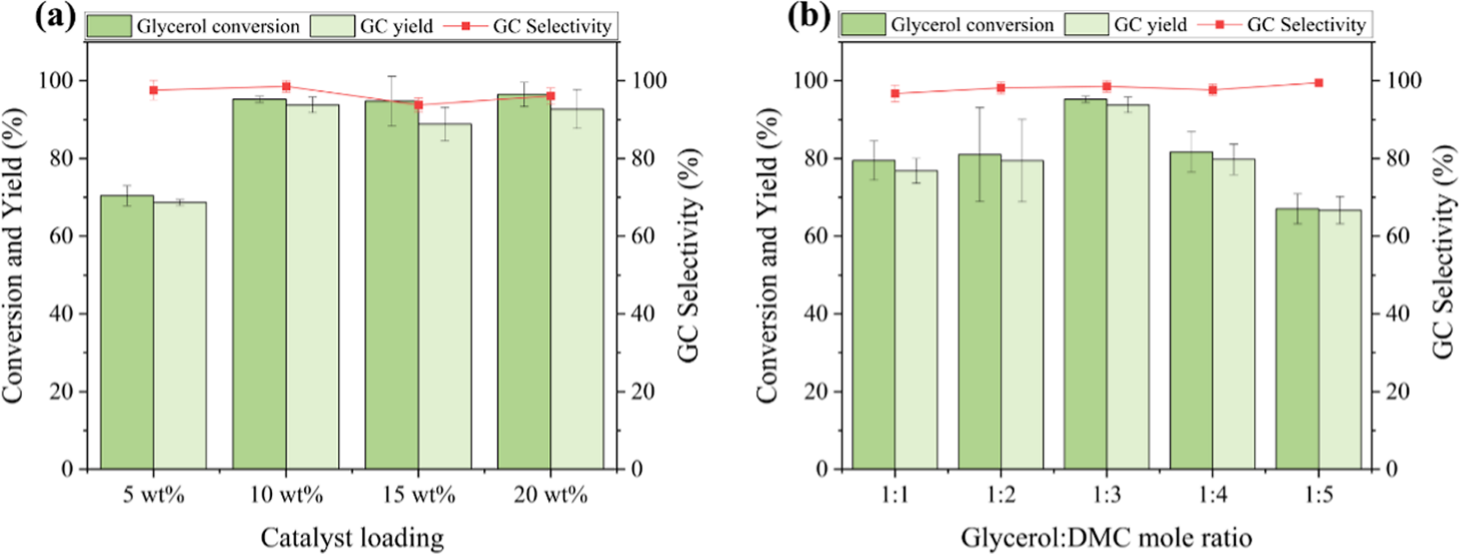
Glycerol conversion, glycerol carbonate selectivity, and yield of 40 wt % CaO–CeO_2_ catalyst with (a) different catalyst loadings and (b) different glycerol to DMC mole ratios.

### 3.4 Effect of Glycerol-to-DMC Mole Ratio

The mole ratio of reactants, glycerol to dimethyl carbonate (GLY: DMC), is a critical factor influencing both the glycerol conversion and the GC yield. In this study, the mole ratio was varied from 1:1 to 1:5 to assess its impact on GC production, as shown in Figure 7b. Initially, increasing the mole ratio of GLY to DMC from 1:1 to 1:3 led to a corresponding increase in the GC yield from 77% to 94%, respectively. Further increase in the mole ratio of GLY to DMC to 1:5 resulted in a decline in the GC yield to 67%. This reduction may be attributed to the increased immiscibility of the reactants. Since glycerol is hydrophilic and DMC is hydrophobic, a higher concentration of DMC in the reaction medium led to phase separation, which hinders mass transfer and reduces the overall reaction efficiency.^29,30^

### 3.5 Kinetic Model Development

The Weisz–Prater criterion (*C*_WP_) was determined to confirm the absence of intraparticle diffusion resistance. This parameter represents the ratio of the intrinsic reaction rate to the intraparticle diffusion rate and serves as an indicator of whether mass transfer limitations affect the reaction kinetics. *C*_WP_ is determined using the observed reaction rate, particle radius (*R*_p_), effective diffusivity of the limiting reactant (*D*_e_), and the concentration of the reactant at the particle’s external surface. The dimensionless Weisz–Prater criterion, *C*_WP_, can be calculated by using eq 3 below.

3 $$C_{WP}=\frac{r_{obs}\rho_{c}R_{p}^{2}}{D_{e}C_{G}}$$

where *r*_obs_ = observed reaction rate (mol kg^–1^ s^–1^), *R*_p_ = catalyst particle radius (m), ρ_c_ = catalyst density (kg m^–3^), *D*_e_ = effective diffusivity between glycerol and DMC (m^2^ s^–1^), and *C*_G_ = bulk liquid glycerol concentration (mol m^–3^).

The effective diffusivity (*D*_e_) was determined with the following equation

4 $$D_{e}=\frac{\varepsilon }{\tau }D_{AB}$$

where ε = porosity of catalyst, τ = tortuosity, and *D*_AB_ = diffusion coefficient between glycerol and DMC (m^2^ s^–1^) calculated by the Wilke–Chang equation

5 $$D_{AB}=\frac{7.4\times 10^{-8}\times {\left(\phi M_{A}\right)}^{1/2}T}{\eta \times V_{B}^{0.6}}$$

Finally, *r*_obs_ is

6 $$r_{obs}=\frac{N_{A0}-N_{At}}{t}\times \frac{W_{c}}{\rho_{c}}$$

where *N*_A0_ and *N*_At_ = moles of glycerol at initial and *t* time (mol), respectively. *t* = reaction time (s), *W*_c_ = mass of catalyst (g).

Conservative estimations for the porosity and tortuosity of the catalyst were 0.05 and 4.41, respectively. The Weisz–Prater Criterion was calculated at different temperatures, as shown in Table 3. The *C*_WP_ values were much below 1, showing the absence of diffusion limitations and no concentration gradient within the catalyst.

**Table 3:** Calculated Weisz–Prater Criterion at Different Reaction Temperatures

temperature (°C)	*r*_obs_ (mol cm^–3^ s^–1^)	*D*_AB_ (cm^2^ s^–1^)	*C*_WP_
60	1.105 × 10^–6^	2.700 × 10^–5^	5.335 × 10^–7^
70	1.418 × 10^–6^	2.781 × 10^–5^	6.651 × 10^–7^
80	2.172 × 10^–6^	2.862 × 10^–5^	9.897 × 10^–7^
90	2.592 × 10^–6^	2.943 × 10^–5^	1.489 × 10^–6^

In synthesizing glycerol carbonate, a second-order irreversible kinetic model was developed based on the rate of glycerol conversion. Also, in a prior article, the second-order irreversible kinetic model was developed and validated to describe the effective transesterification of glycerol with DMC.^31^ Since the side reaction involving DMC was found to be negligible, only the transesterification of glycerol was considered in the kinetic analysis. Consequently, the kinetic model for glycerol transesterification was simplified to represent the overall reaction, as shown in eq 7.

7 $$Glycerol+DMC\overset{k}{\to }glycerol\;carbonate+2MeOH$$

The surface reaction of the adsorbed species on the catalyst was assumed to be the rate-determining step. The surface reactions were considered irreversible due to the excess DMC used relative to glycerol. As a result, the kinetic model can be represented by eq 8, where A and B represent glycerol and DMC, respectively.

8 $$R_{A}=\frac{dC_{A}}{dt}=kC_{A}C_{B}$$

where *k* is an irreversible second-order rate constant, *C*_A_ is the glycerol concentration, and *C*_B_ is the concentration of DMC. According to Teng et al.^31^ eq 8 can be written in terms of fractional conversion in the form of eq 9.

9 $$\frac{dX_{A}}{dt}=kC_{A0}\left(1-X_{A}\right)\left(M-X_{A}\right)$$

This, upon integration, leads to

10 $$\frac{1}{C_{A0}\left(M-1\right)}\;\ln\frac{\left(M-X_{A}\right)}{\left(1-X_{A}\right)}=kt+C$$

where *X*_A_ is the conversion of glycerol. *M* = *C*_B0_/*C*_A0_, and *C*_A0_ and *C*_B0_ are the initial molar concentrations of glycerol and DMC, respectively.

Equation 10 can be written in terms of the linear equation, as below

11 Y(t)=kt+C

where

$$Y\left(t\right)=\frac{1}{C_{A0}\left(M-1\right)}\ln\frac{\left(M-X_{A}\right)}{\left(1-X_{A}\right)}$$

To validate the reaction kinetics, the irreversible second-order kinetic model was tested by plotting eq 10.

The reaction temperature plays a crucial role in influencing the transesterification process. Typically, as the temperature increases, the reaction rate accelerates, leading to a higher GC yield. To investigate this effect, the reaction temperature was varied between 60 and 90 °C, using a 10 wt % catalyst loading and a GLY to DMC mole ratio of 1:3, with a reaction time of 3 h. A significant increase in both glycerol conversion (Figure 8a) and GC yield (Figure 8b) was observed as the temperature increased from 60 to 90 °C. At 90 °C, a glycerol conversion of 96% was achieved within 1 h, which further increased to 99% after 3 h. A higher reaction temperature increases the frequency of molecular collisions, thereby accelerating the reaction rate. Since the transesterification of DMC with glycerol is an exothermic reaction,^32^ increasing the temperature may shift the equilibrium position favorably, resulting in improved glycerol conversion and higher GC yield.^33^ Similarly, the GC concentration increased significantly to 4.10 mol L^–1^ at 90 °C, followed by a slight increase to 4.70 mol L^–1^ after 3 h of reaction time.

**Figure 8:**
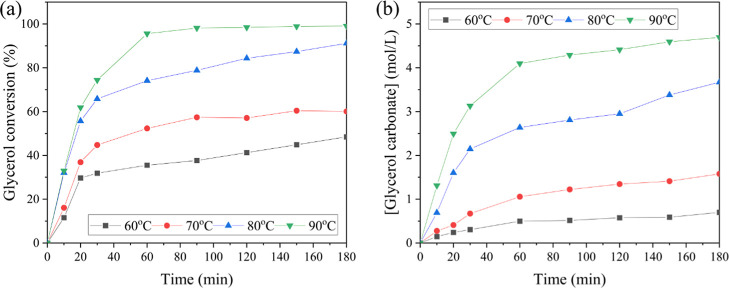
(a) Reaction time profile for glycerol conversion at different temperatures and (b) reaction time profile for glycerol carbonate production at different temperatures.

### 3.6 Rate Constants and Activation Energy

The conformity of the reaction kinetics with an irreversible second-order kinetic model was evaluated by plotting eq 10 with the results presented in Figure 9. The slope and intercept of the trend lines in the figure correspond to the forward reaction rate constant and the model integration constant, respectively. In addition, the *R* ^2^ values of the trend lines indicate the confidence levels of the reaction conformity with the proposed kinetic model. The reaction showed confidence levels in the range of 95–99%, demonstrating a strong agreement with the second-order kinetic model.

**Figure 9:**
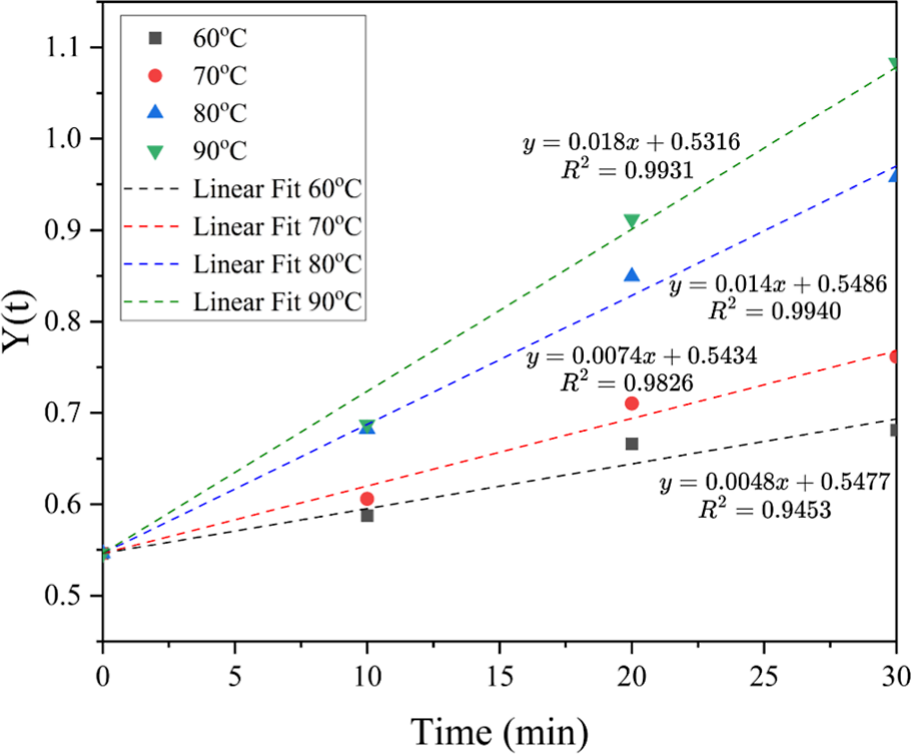
Plot of *Y*(*t*) against time at a different temperature is achieved from the irreversible second-order kinetic model.

The rate constants at different reaction temperatures are summarized in Table 4, determined from the slopes of the trendlines presented in Figure 9 and calculated using eq 4. The lowest rate constant, 0.0048 L mol^–1^ min^–1^, was obtained at 60 °C, while the highest, 0.0180 L mol^–1^ min^–1^, was observed at 90 °C. This indicated that increasing the temperature enhances the reaction rate, thereby reducing the time required to achieve a maximum product yield. These findings align with previously discussed equilibrium times at various temperatures. The activation energy of the reaction was calculated using the Arrhenius equation, as shown in eq 12. The data in Table 4 clearly demonstrate that the apparent reaction rate constant increases with temperature from 60 to 90 °C, indicating that equilibrium is reached more rapidly at higher temperatures. The activation energy for the transesterification reaction, calculated from the Arrhenius plot in Figure 10, was found to be 46.9 kJ mol^–1^. Literature values for activation energy for this reaction typically range between 28.4 and 52.5 kJ mol^–1^.^32,34–36^ These results confirm that the reaction proceeded without mass transfer limitations.

12 $$k=A\;\mathrm{Exp}\left(-\frac{E_{a}}{RT}\right)$$

where *E*_a_ is the activation energy and *A* is the frequency factor. In the plot of ln *k* versus 1/*T*, the slope and the intercept of the trend line denote the value of *E*_a_/*R* and *A*, respectively.

**Table 4:** Rate Constants for the Transesterification

temperature (°C)	rate constant, *k* (L mol^–1^ s^–1^)
60	0.0048
70	0.0074
80	0.0140
90	0.0180

**Figure 10:**
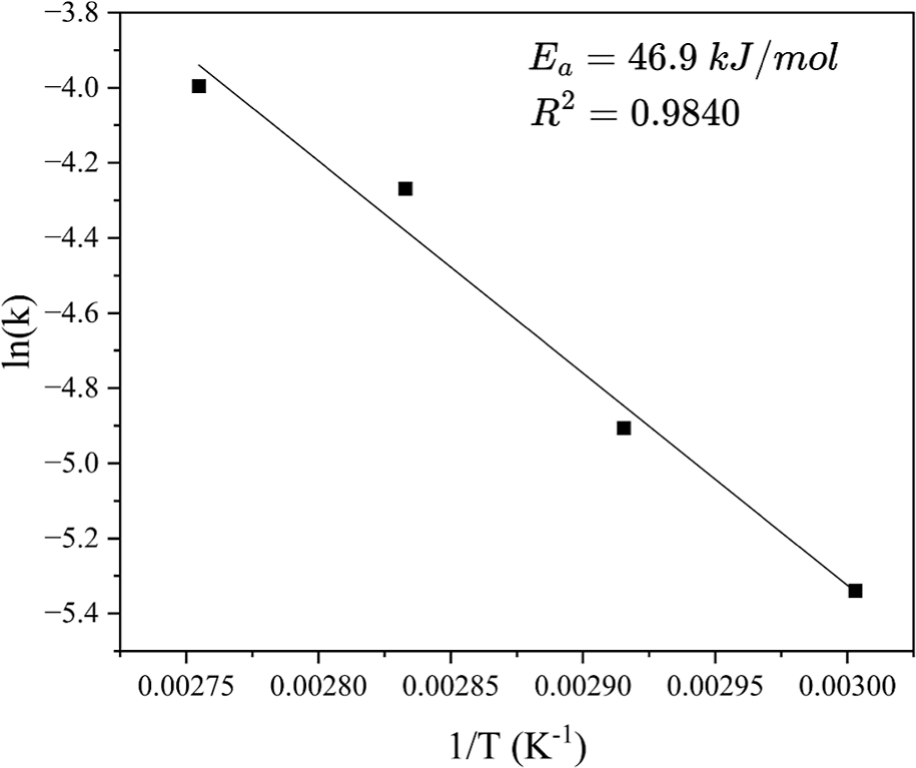
Arrhenius plot for the irreversible second-order kinetic model for the transesterification of glycerol with DMC.

### 3.7 Catalyst Reusability

The consecutive reaction cycles were performed to evaluate the stability and reusability of the catalysts with the results shown in Figure 11. The catalyst showed sustained activity, maintaining high glycerol conversion and GC yield over four reaction cycles. The spent catalysts were characterized using XRD and FTIR spectroscopy to evaluate the structural changes in the surface CaO species. As shown in Figure 12a, XRD patterns of the used 40 wt % CaO–CeO_2_ revealed peaks corresponding to CaCO_3_ at 17° and Ca­(OH)_2_ at 35° and 52°. Also, some CaO peaks present in the fresh catalyst were absent in the used catalyst. According to Praikaew et al.,^21^ transformation of CaO can occur via the formation of Ca­(OH)_2_ and CaCO_3_ species during the reaction process. The formation of CaCO_3_ may be influenced by an excess of DMC relative to glycerol, potentially facilitating a condensation reaction between glycerol and CaO. This leads to the in situ generation of water and water-derived species. Consequently, CaO can undergo further reaction with DMC and water, producing methanol and CaCO_3_. Moreover, SEM analysis (Figure 5e) indicated that the CaCO_3_ formed during the reaction existed as an amorphous layer on the catalyst surface, altering its morphology to smoother, rounded, and irregularly shaped particles.^20^ When the used catalyst was recovered and calcined at 800 °C for 3 h, it converted CaCO_3_ and Ca­(OH)_2_ back into CaO. The XRD patterns of the used catalyst calcined at 800 °C were similar to those of the fresh catalyst, confirming the regeneration of CaO. FTIR analysis (Figure 12b) further validated this transformation. After the reaction, a peak at 875 cm^–1^ became sharper compared to the fresh and calcined catalysts, which can be attributed to the out-of-plane stretching vibrations of the carbonate group (CO_3_). Additionally, the band at 1397 cm^–1^ exhibited an increased intensity after the reaction, corresponding to the asymmetric stretching of the C–O bond in the carbonate group. Additional bands at approximately 1489 cm^–1^ appeared, which could be associated with C–O stretching in esters or alcohols and alkyl group stretching, respectively. Notably, the band at 1043 cm^–1^ is characteristic to C–O stretching vibrations in the methoxide (−OCH_3_) group of calcium methoxide, indicating that CaO initially reacts with methanol to form calcium methoxide, which then hydrolyzes to Ca­(OH)_2_ and subsequently might convert to CaCO_3_ upon exposure to moisture and CO_2_.^21^ These findings confirm that calcination effectively removes surface impurities and facilitates the regeneration of CaO, thereby maintaining catalytic activity and glycerol conversion efficiency.

**Figure 11:**
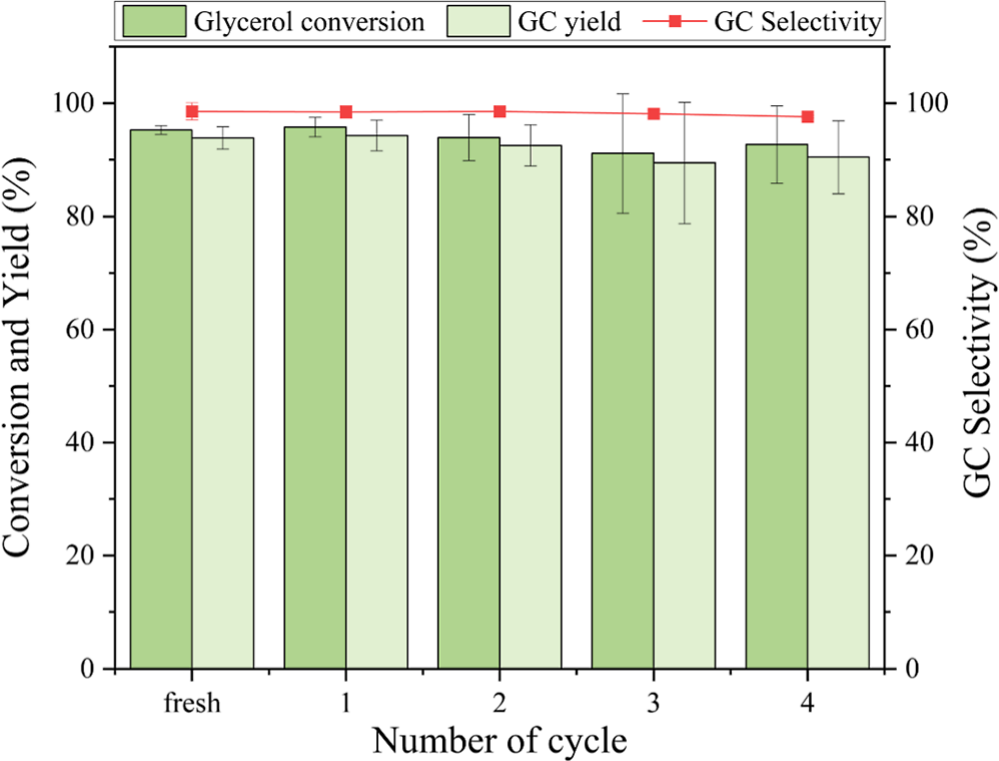
Effect of 40 wt % CaO–CeO_2_ reusability on glycerol conversion and product yield.

**Figure 12:**
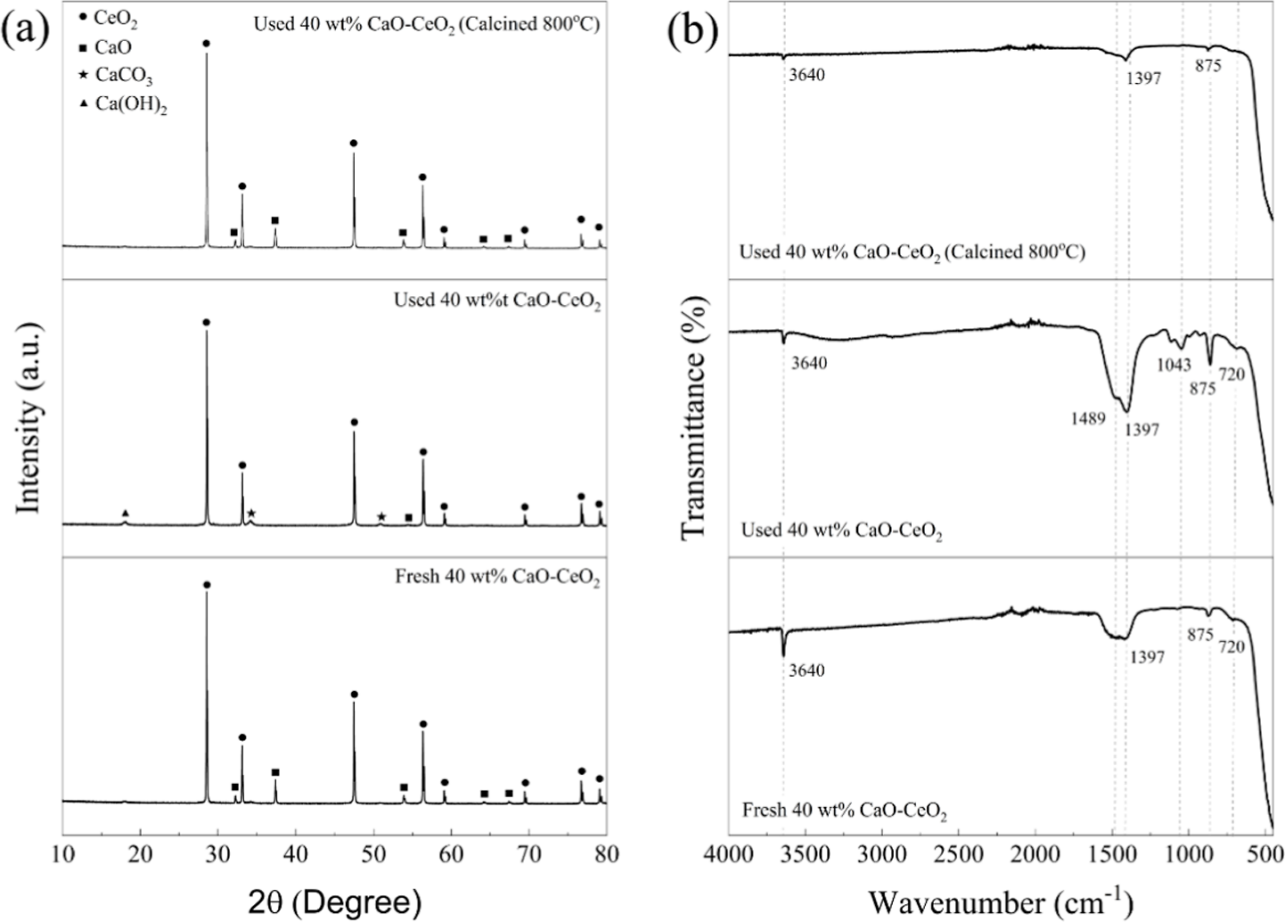
(a) X-ray diffraction patterns of used 40 wt % CaO–CeO_2_ catalysts before and after calcination, and (b) FTIR spectra of used 40 wt % CaO–CeO_2_ catalysts before and after calcination.

### 3.8 Catalyst Stability

The leaching test was performed to investigate the catalyst stability by the hot filtration method. After 30 min of reaction, the catalyst was removed from the reaction mixture by filtration, and the resulting filtrate was continued without catalyst under the same reaction conditions for an additional 60 min. As illustrated in Figure 13a, no further conversion of glycerol was observed following the removal of the 40 wt % CaO–CeO_2_ catalyst. This result indicates that the catalyst remains largely heterogeneous during the reaction, suggesting minimal leaching. The presence of CeO_2_ enhanced the structural integrity of the catalyst, acting as a stabilizing support and inhibiting the dissolution of the active CaO species. In contrast, when pure CaO was employed as the catalyst, the reaction continued gradually even after its removal, achieving 70% glycerol conversion at 90 min of reaction time—comparable to that achieved with the catalyst present throughout the reaction period (Figure 13b). This observation implies partial leaching of the active species from the pure CaO catalyst into the reaction medium.

**Figure 13:**
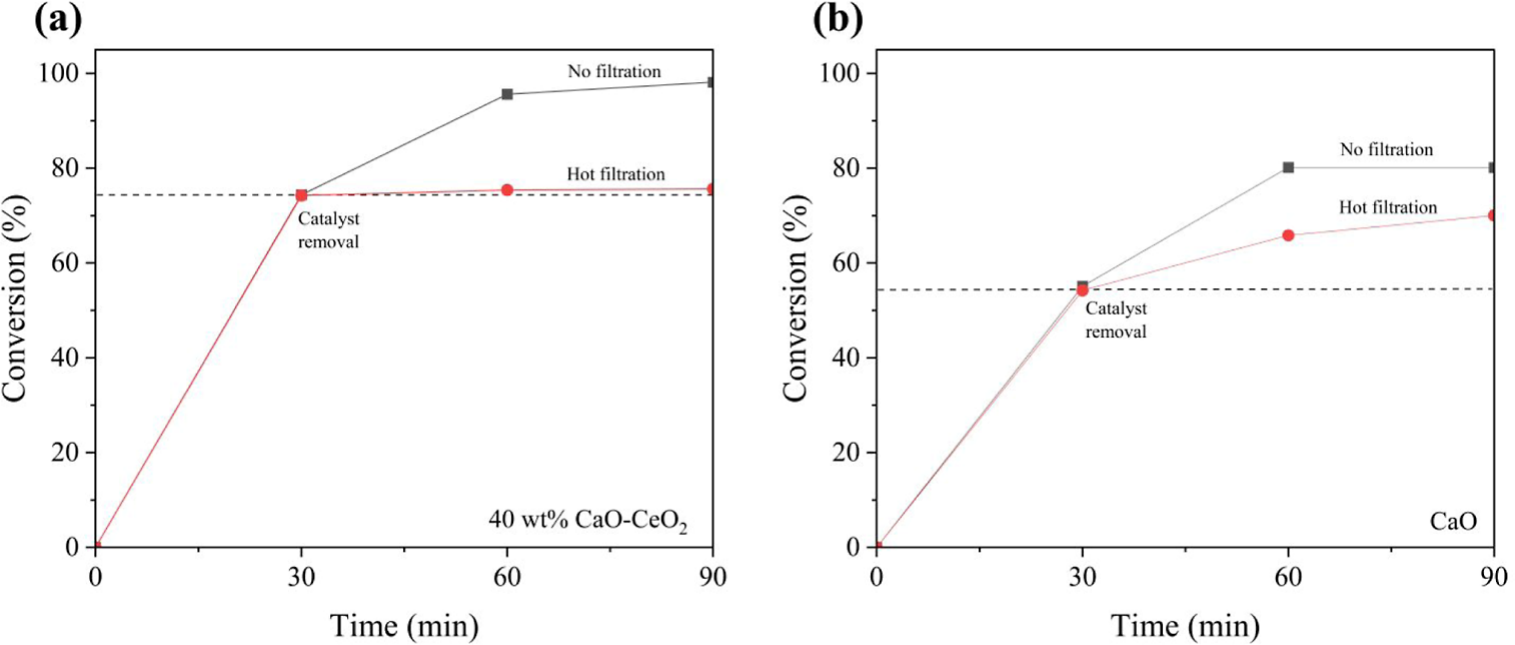
Hot filtration method for the evaluation of catalyst leaching: (a) 40 wt % CaO–CeO_2_ catalysts and (b) CaO catalyst.

### 3.9 Reaction Mechanism

A plausible reaction mechanism for the transesterification of glycerol with dimethyl carbonate (DMC) over heterogeneous base catalysts, including CaO and Ca-doped metal oxides, has been widely investigated in previous studies.^11,20,21,28,33,37^ Herein, we propose the mechanism for glycerol carbonate formation on the surface of the CaO–CeO_2_ catalyst via synergistic interaction, as illustrated in Scheme 1. Catalyst characterization, particularly CO_2_-TPD along with catalytic activity tests, indicate that the total basicity of CaO–CeO_2_ mixed oxides increases with CaO doping, correlating with a higher GC yield. Scheme 1 highlights the synergistic interaction between basic active sites of the O^2–^ anions, Ca^2+^–O^2–^ pairs, and oxygen vacancies in the CeO_2_ matrix at the CaO–CeO_2_ interface. Initially, glycerol adsorbs onto the basic active sites of the catalyst, specifically, the O^2–^ anions and Ca^2+^–O^2–^ pairs, while the carbonyl group of DMC interacts with oxygen vacancies in CeO_2_. The basic sites of O^2–^ and Ca^2+^–O^2–^ activate glycerol, forming a glyceroxide anion via hydrogen bonding. Simultaneously, the electron-rich carbonyl oxygen in DMC interacts with Ce^3+^ sites at the oxygen vacancies. The glyceroxide anion at the CaO–CeO_2_ interface then nucleophilically attacks the carbonyl group of DMC adsorbed on Ce^3+^ sites, forming a methyl glyceryl carbonate intermediate and releasing a methoxide anion, which subsequently converts into methanol. In the final step, an adjacent hydroxyl group’s activated oxygen attacks the carbonyl carbon, facilitating cyclization and yielding glycerol carbonate, with methanol as a byproduct.

**Scheme 1:**
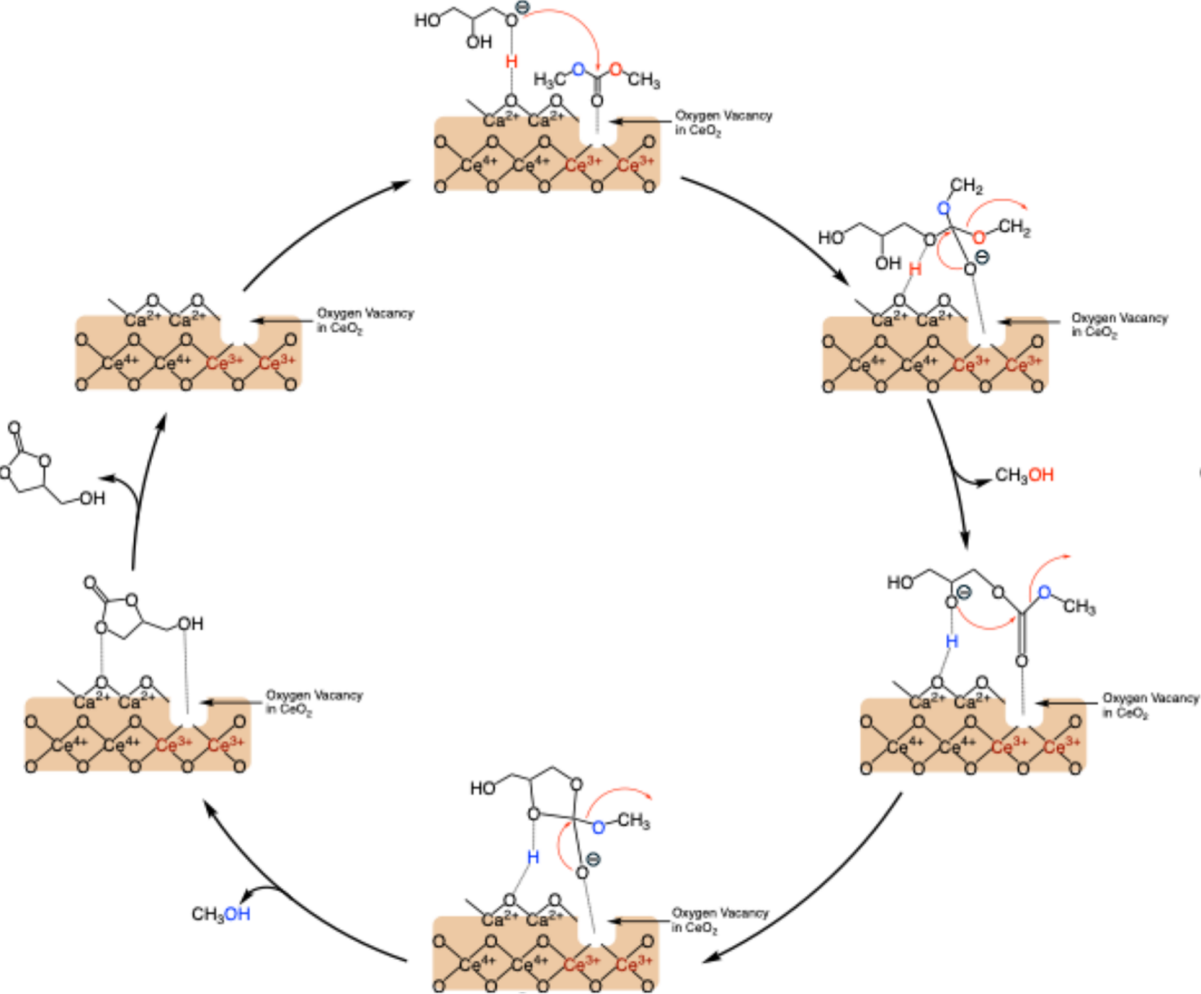
Plausible Mechanism of Transesterification between Glycerol with DMC over CaO–CeO_2_ Catalyst

### 3.10 Activity Comparison

The catalytic performance of the 40 wt % CaO–CeO_2_ catalyst was compared with that of various heterogeneous catalysts previously reported for the transesterification of glycerol with dimethyl carbonate (DMC), as summarized in Table 5. Under optimized conditions (90 °C, 10 wt % catalyst loading, DMC/glycerol molar ratio of 3, and 90 min of reaction time), the CaO–CeO_2_ catalyst achieved an outstanding glycerol carbonate (GC) yield of 98%. This yield exceeds those reported for conventional CaO (70%)^38^ and modified CaO systems such as 0.5 Ben/CaO (82%)^38^ under similar conditions, requiring longer reaction times and delivering significantly lower conversions.

**Table 5:** Comparison of 40 wt % CaO–CeO_2_ Catalyst with Other Reported Catalysts for the Synthesis of Glycerol Carbonate

catalyst	temperature (°C)	catalyst dosage (wt %)	DMC/Gly	time (min)	GC yield (%)	ref
CaO	95	5%	5	120	70.0	^38^
0.5 Ben/CaO	95	5%	5	120	82.0	^38^
15 wt % Na_2_CO_3_–Cs-800	75	3%	5	90	96.0	^20^
5 wt % Co/MCM-41	90	6%	3	120	94.1	^36^
MMO-Cu_15_Zn_15_	85	7.5%	2	270	85.1	^44^
Mg/ZnO	80	3%	4	120	96.5	^41^
6MgO/g-C_3_N_4_	80	5%	3	240	97.1	^39^
Mg_3_Ce_1_	90	15%	5	90	86.0	^15^
Mg_2.4_Al_1.0_Cu_0.6_	90	15%	5	90	91.2	^45^
Mg_3_Al_1_Zr_1_	75	10%	5	90	94.0	^43^
1:2 CuO/ZnO/MnO_2_	90	3%	5	90	97.0	^42^
CoFe_2_O_4_/CaO–ZnO	85	5%	5	150	97.7	^40^
40 wt % CaO–CeO_2_	90	10%	3	90	98.1	in this work

Other catalysts have demonstrated competitive yields, although with compromises in reaction time, temperature, and catalyst complexity. For example, 6MgO/g-C_3_N_4_ and CoFe_2_O_4_/CaO–ZnO achieved yields of 97% and 98%, respectively, but required prolonged reaction times of 240 and 150 min^39,40^ Likewise, Mg/ZnO and Na_2_CO_3_–Cs-800 catalysts yielded 97% and 96%, respectively, yet required longer durations or specific synthetic modifications.^20,41^ Notably, multicomponent systems such as 1:2 CuO/ZnO/MnO_2_ (97%) and Mg_3_AlZr_1_ (94%) also exhibited high performance, however, involving more complex formulations that may limit their scalability.^42,43^

Conversely, the CaO–CeO_2_ catalyst provides an advantageous combination of high activity, short reaction time, and operational simplicity. Moreover, its preparation via a green, solvent-free method and using earth-abundant components are economically and environmentally sustainable options for industrial implementation in glycerol carbonate production.

## 4 Conclusion

A sustainable approach for synthesizing glycerol carbonate from glycerol and dimethyl carbonate was demonstrated using a supported CaO catalyst. Cerium oxide (CeO_2_) served as a support material, enhancing the catalyst stability and activity. Among the catalyst compositions tested, 40 wt % CaO–CeO_2_ exhibited the highest catalytic performance, achieving a glycerol carbonate yield exceeding 95% at 90 °C. The catalyst showed excellent stability and reusability, maintaining its high performance over four cycles. A comprehensive kinetic model was developed based on irreversible second-order kinetics. The activation energy for the transesterification reaction, determined from an Arrhenius plot, was calculated as 46.9 kJ mol^–1^, confirming that the reaction proceeded within the kinetic regime rather than being diffusion-limited. Overall, this study highlights the effectiveness of mechanochemically prepared CaO–CeO_2_ catalysts in enabling the green synthesis of glycerol carbonate, offering a promising route for a sustainable circular economic process.

## Abbreviations

DMC: dimethyl carbonate
Eqn: equation
GC: glycerol carbonate
GLY: glycerol
